# Radiomics analysis based on lumbar spine CT to detect osteoporosis

**DOI:** 10.1007/s00330-022-08805-4

**Published:** 2022-04-30

**Authors:** Yan-Wei Jiang, Xiong-Jie Xu, Rui Wang, Chun-Mei Chen

**Affiliations:** Department of Neurosurgery, Fujian Medical University Union Hospital, No. 29, Xin Quan Road, Fuzhou City, 350001 Fujian Province China

**Keywords:** Radiomics, Vertebral body, ROC curve, Osteoporosis

## Abstract

**Objectives:**

Undiagnosed osteoporosis may lead to severe complications after spinal surgery. This study aimed to construct and validate a radiomic signature based on CT scans to screen for lumbar spine osteoporosis.

**Methods:**

Using a stratified random sample method, 386 vertebral bodies were randomly divided into a training set (*n* = 270) and a test set (*n* = 116). A total of 1040 radiomics features were automatically retracted from lumbar spine CT scans using the 3D slicer pyradiomics module, and a radiomic signature was created. The sensitivity, specificity, accuracy, and area under the receiver operating characteristic curve (AUC) of the Hounsfield and radiomics signature models were calculated. The AUCs of the two models were compared using the DeLong test. Their clinical usefulness was assessed using a decision curve analysis.

**Results:**

Twelve features were chosen to establish the radiomic signature. The AUCs of the radiomics signature and Hounsfield models were 0.96 and 0.88 in the training set and 0.92 and 0.84 in the test set, respectively. According to the DeLong test, the AUCs of the two models were significantly different (*p* < 0.05). The radiomics signature model indicated a higher overall net benefit than the Hounsfield model, as determined by decision curve analysis.

**Conclusions:**

The CT-based radiomic signature can differentiate patients with/without osteoporosis prior to lumbar spinal surgery. Without additional medical cost and radiation exposure, the radiomics method may provide valuable information facilitating surgical decision-making.

**Key Points:**

• *The goal of the study was to evaluate the efficacy of a radiomics signature model based on routine preoperative lumbar spine CT scans in screening osteoporosis.*

• *The radiomics signature model demonstrated excellent prediction performance in both the training and test sets.*

• *This radiomics method may provide valuable information and facilitate surgical decision-making without additional medical costs and radiation exposure.*

**Supplementary Information:**

The online version contains supplementary material available at 10.1007/s00330-022-08805-4.

## Introduction

Osteoporosis is a bone condition characterized by a decrease in bone density and worsening quality of bone microarchitecture. The most common screening modality for this disease is dual-energy X-ray absorptiometry (DEXA). However, the screening rate for osteoporosis remains low. DEXA is not routinely performed before spinal surgery in clinical practice due to its high medical costs and radiation exposure [1–3]. Osteoporosis may lead to severe complications after spinal surgery, such as fusion failure [1]. An increased screening rate may reduce osteoporosis-related complications.

CT is a common diagnostic imaging modality for the preoperative assessment in lumbar spine surgery. Using CT scans to measure vertebral HU values has shown promise in previous studies. A low HU value may be associated with complications, such as pseudoarthrosis after lumbar fusion surgery [4]. Schreiber et al [5] found that the HU value obtained from a routine CT scan significantly correlates with DEXA results, and Zou et al [6] found that the HU value is a good predictor of pedicle screw loosening after lumbar fixation. Furthermore, HU measurement allows clinicians to screen for osteoporosis preoperatively without additional radiation exposure and medical costs [7]. However, HU measurements did not include the cortical bone in the method described; therefore, in the real world, this assessment may lose a significant amount of information needed to assess bone quality [5]. The mean HU value of the vertebral region of interest (ROI) may differ according to different observers. Cancellous bone is heterogeneous; therefore, HU based on axial sections may not accurately reflect the bone quality [2, 5, 8, 9].

Radiomics features are large amounts (200+) of quantitative features mathematically extracted from medical images, believed to reflect intra-region heterogeneity [10]. Radiomics hypothesizes that these quantitative features may provide unknown information related to specific diseases [10–13]. In oncology, radiomics is mainly used for the non-invasive estimation of the clinical diagnosis and prognosis [14]. Recently, Wu et al [15] applied a CT-based radiomics method to predict the outcome of COVID-19 patients, and Mookiah et al [16] used the radiomics features derived from multidetector CT to evaluate the trabecular bone quality and osteoporosis in the laboratory. However, no study has assessed bone quality based on the high-throughput radiomic features extracted from routing CT scans. In this study, radiomic features were extracted from the 3D segmentation of the entire vertebral body, comprising both the cancellous and cortical bones to determine the efficacy of a radiomics model based on routine preoperative lumbar spine CT scans in screening for osteoporosis.

## Materials and methods

### Study participants

The local Ethics Committee of our institution approved this retrospective study (approval no. 2021KJCX038). Consecutive patients were included by reviewing the database of our department from January 2020 to June 2021. We identified 99 patients who had lumbar spine CT and DEXA within 14 days. Exclusion criteria were (1) related vertebral body tumor, (2) ankylosing spondylitis, and (3) diffuse idiopathic skeletal hyperostosis.

**Fig. 1:**
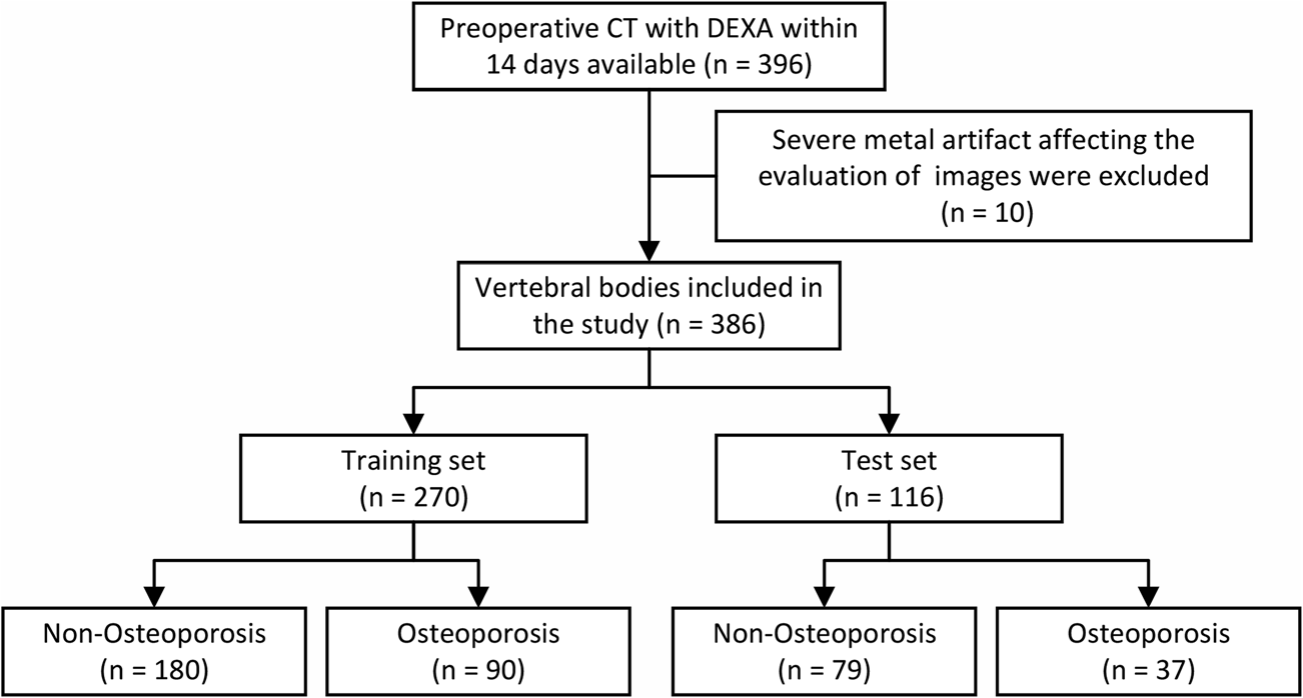
Flowchart for selecting the study population

Using DEXA, 34 of the 99 patients in the study were diagnosed with osteoporosis. For each patient, the L1–L4 vertebral bodies were chosen for the analysis; thus, a total of 396 vertebral bodies were initially included in this study. However, 10 of these were excluded for severe metal artifacts affecting the image evaluation. Among the 386 vertebral bodies considered, 127 (32.9%) had osteoporosis, based on DEXA results. These vertebral bodies were randomly divided into two groups at a 7:3 ratio using a stratified random sampling method. Thus, the training cohort included 270 participants while the test cohort included 116. A pipeline depicting patient selection is displayed in Fig. 1. The clinical characteristics were obtained from the patient records in our hospital.

### Image acquisition

All CT images and DEXA results were collected from our department’s picture archiving and communication system (PACS). Each patient underwent a lumbar spine CT scan using a helical 64-channel CT scanner. The following CT scanning parameters were used: tube voltage of 120 kVp, tube current of 300 mA, and slice thickness of 1.25 mm at 0.625-mm intervals. The DEXA scans were performed in each patient’s spine and hip, and the lumbar vertebrae were divided into two groups based on diagnosis: osteoporosis and non-osteoporosis groups.

### Image segmentation, radiomic feature extraction, and HU measurement

**Fig. 2:**
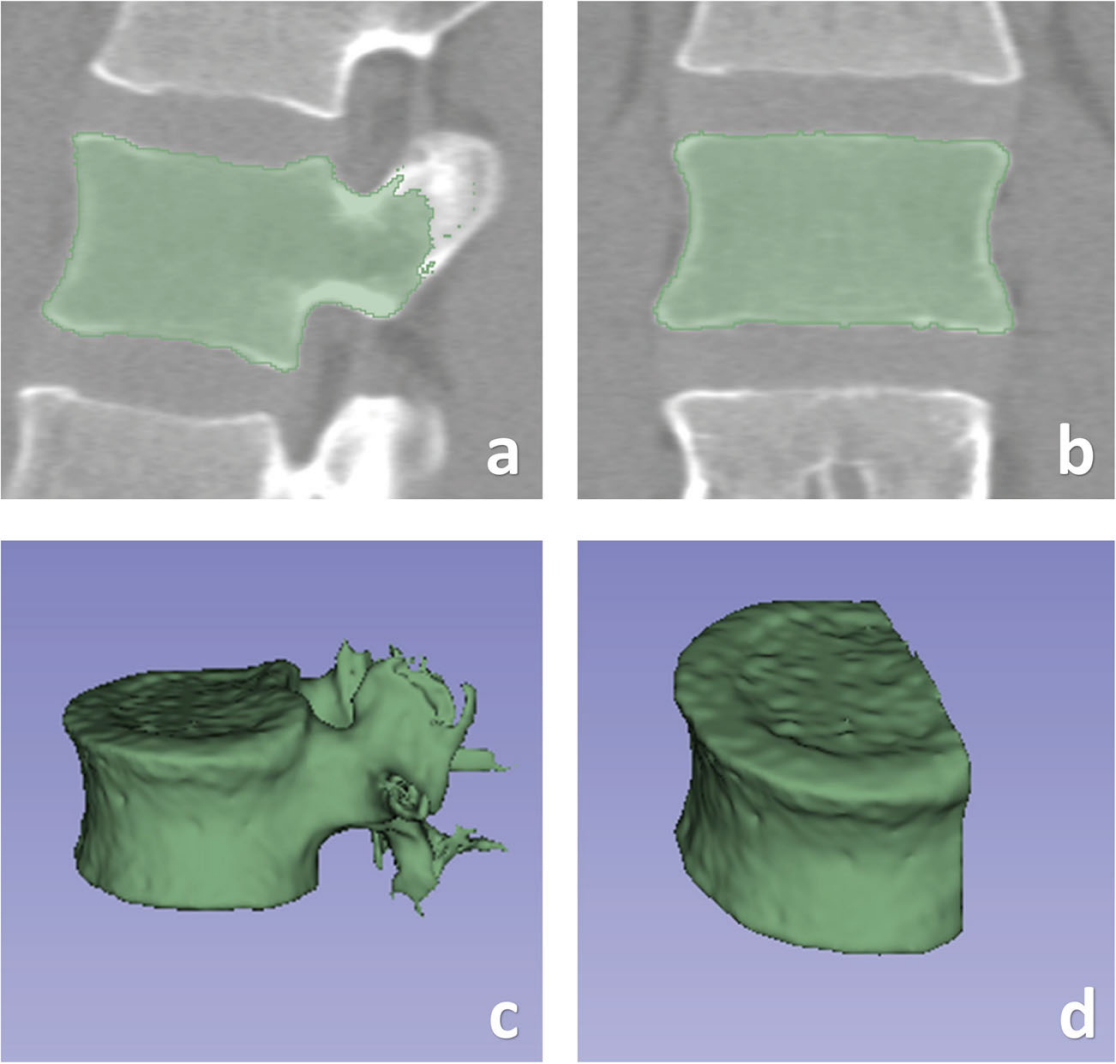
Semi-automatic segmentation of the vertebral body. **a** Segmentation on a sagittal slice. **b** Segmentation on a coronal slice. **c** 3D volumetric reconstruction. **d** The posterior potion of the vertebral body was excluded to reduce the influence of the vertebral pedicle

3D Slicer (\https://www.slicer.org), a free and open-source software, was used for the 3D segmentation of the vertebral bodies. The images were segmented semi-automatically in the vertebral region using the segmentation threshold and seed growing module. The posterior portion of the vertebral body was removed to alleviate the influence of the vertebral pedicle. An example of segmentation is presented in Fig. 2.

Among the features obtained from the segmented vertebral bodies, 1040 were automatically extracted by applying the 3D Slicer pyradiomics module. Resampling and *z*-score normalization were performed to guarantee the repeatability of the feature extraction. The extracted radiomic features are provided in Supplementary material S1.

The HU values were measured from PACS data using a technique previously described [5]. The ROIs were positioned on the midbody axial CT image for each vertebra. A ROI was defined as a single maximally sized ellipse including the maximum trabecular bone while avoiding the cortical bone of the vertebral body. The measurement was performed in one slice per vertebra. The mean HU value obtained was used for further investigation.

A total of 36 vertebrae were randomly selected to evaluate the reliability of HU measurements and radiomic features. One month after the first author (Y.W.J.) measured the HU values, the radiomic features of the vertebrae were extracted. The co-author (R.W.) repeated the measurement and extraction process to evaluate the inter-observer reliability. The two observers were blinded to the clinical information when performing the measurements. The intraclass correlation coefficient (ICC) was used to determine the intra- and inter-observer variability.

### Establishment of radiomic signature and HU models

Dimension reduction was performed before the radiomic signature construction to eliminate overfitting or any type of bias in our radiomic model. First, the radiomic features with ICCs (both in inter- and intra-observer classes) > 0.75 were regarded as reliable and selected. The minimum redundancy-maximum relevance (mRMR) algorithm was used to determine the relevance and redundancy of each feature. The 30 features with the highest-ranked mRMR were selected to construct the model. The optimal feature subset was then chosen using the least absolute shrinkage and selection operator (LASSO) regression model (a selection method for linear regression). This method can shrink the regression coefficients and reduce some to zero; the remaining coefficients were applied to construct the final model. Tenfold cross-validation was used to tune the regularization parameter (*λ*). Using the following formula, the radiomics score (Rad score) of each vertebral body was calculated.

$$ Rad\  score={\sum}_{i=0}^n\mathrm{C}i\times \mathrm{X}i+b $$

where X*i* represents the *i*th selected feature, *Ci* is the corresponding feature coefficient, and *b* is the intercept.

The radiomic signature model was constructed using the radiomic features extracted from the training set. The model’s capability to identify osteoporosis was assessed in both the training and test sets. In contrast, a ROC curve based on HU values was used to establish the separation criteria between osteoporosis and non-osteoporosis. Using the ROC curve, the most appropriate HU cutoff value was determined by Youden’s index. A logistic regression analysis based on HU was also conducted.

### Evaluation of the HU and radiomics signature models’ performance

The performance of the HU and radiomic signature models was assessed according to the AUC in both training and test sets. We use decision curve analysis (DCA) to determine the clinical benefit of the two models proposed.

### Statistical analysis

All statistical analyses were conducted using R statistical software (ver. 3.4.2, http:// www.r-project.org). The chi-squared test or Fisher’s exact test was used to compare the categorical data between the two groups. “mRMRe” package in R was used to implement the mRMR algorithm. The “glmnet” package was used to implement the LASSO algorithm. The ROC curves were plotted using the “pROC” package. The DeLong test was then used to compare the ROC curves of the two models. Finally, the “rmda” package was used to perform the DCA. Statistical significance was set at *p* < 0.05.

## Results

### Clinical characteristics

There were no differences in clinical characteristics between the training and test cohorts (Table 1).

**Table 1:** Clinical characteristics in the training and validation sets

Characteristics	Training set (*n* = 270)	Test set (*n* = 116)	*p* value
Diagnosis			0.1049
Osteoporosis	90	37	
Non-osteoporosis	180	79	
Age (y)	60.0 ± 13.1	60.6 ± 12.7	0.7066
Sex			0.4896
Male	123	58	
Female	147	58	
BMI	24.57 ± 3.43	24.16 ± 3.55	0.3016
Hounsfield value	124.13 ± 53.16	125.92 ± 58.59	0.7777

*BMI* body mass index

### Establishment of the radiomics signature

**Fig. 3:**
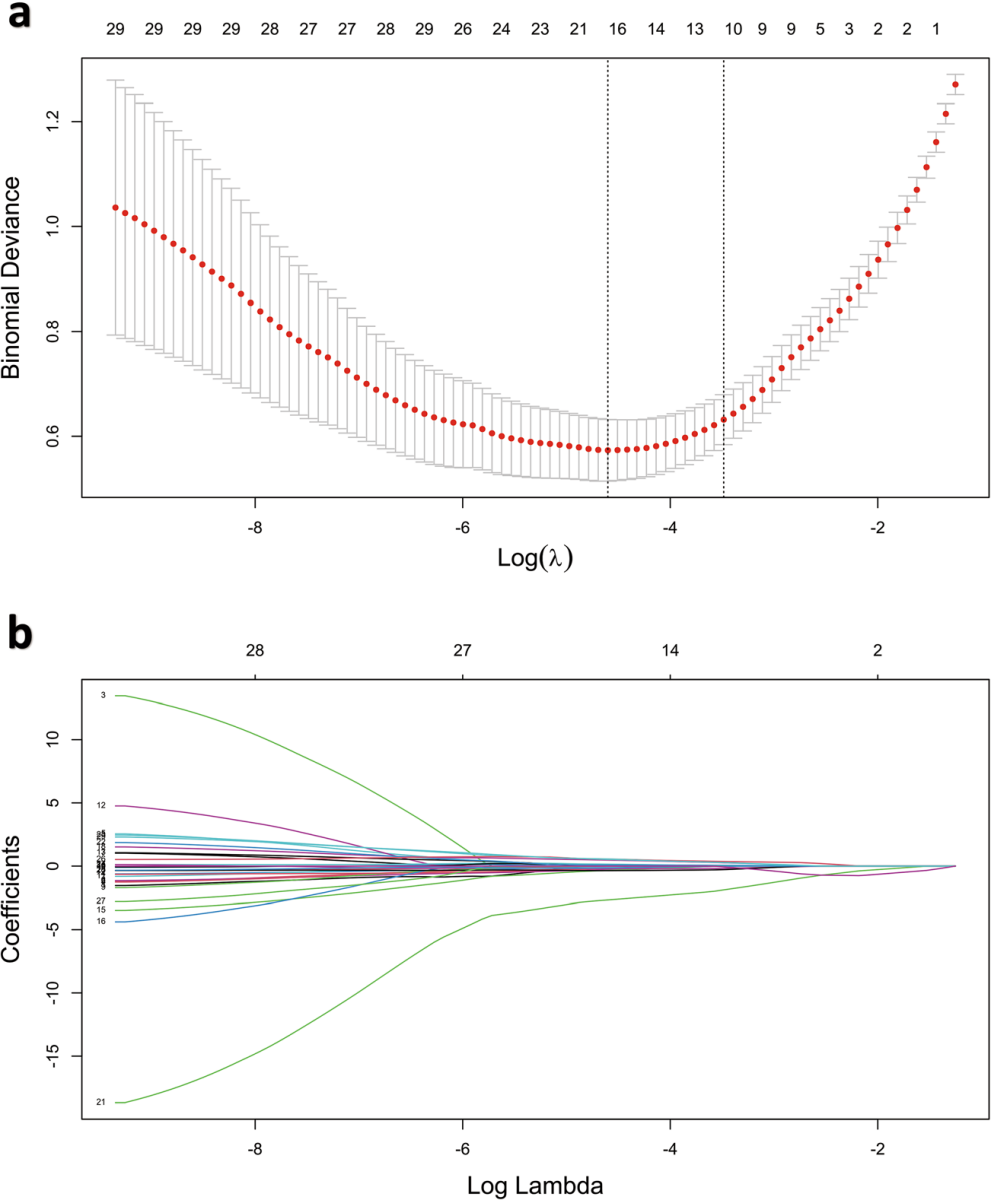
The LASSO regression model was used for the selection of radiomics features. **a** A 10-fold cross-validation was used to select the tuning parameter (*λ*). The y-axis correspond the binomial deviance while the x-axis correspond log (*λ*). The two vertical dotted lines represented the one standard error of the minimum (1-SE) and minimum criteria, respectively, for the specific values. **b** Thirty radiomic features coefficient profile versus the log (*λ*) sequence

From the lumbar spine CT images, 1040 quantitative features were initially extracted, and 846 had an ICC > 0.75. The results of the inter- and intra-observer agreement analyses are provided in Supplementary material S2. Observer 1 performed the segmentation and radiomic extraction on the samples. After applying the mRMR algorithm, 30 features were retained for the LASSO regression method. Tenfold cross-validation was applied to select the tuning parameter (*λ*) in the LASSO regression (Fig. 3). With an optimal *λ* of 0.031 under the 1-SE criteria, the remaining 12 features (Table 2) were used to establish the radiomics signature. As Table 3 shows, the Rad score of the osteoporosis group (0.62 ± 0.18) significantly differed (*p* < 0.05) from that of the non-osteoporosis group (0.18 ± 0.22). The total Rad scores are shown in Supplementary material S3.

**Table 2:** The 12 features chosen for the radiomics model

Image type	Feature class	Feature name	LASSO coefficient (β)
Wavelet-HLH	Firstorder	Kurtosis	−0.01779491
Log-sigma-4-0-mm-3D	Firstorder	Minimum	−0.01415441
Wavelet-LLH	Firstorder	Kurtosis	0.007197711
Log-sigma-5-0-mm-3D	Glszm	LargeAreaLowGrayLevelEmphasis	0.020543574
Wavelet-LLL	Firstorder	10Percentile	−0.188842157
Wavelet-HHL	Ngtdm	Busyness	0.011143668
Wavelet-LLL	Glszm	LargeAreaLowGrayLevelEmphasis	0.029467634
Log-sigma-4-0-mm-3D	Glszm	SmallAreaEmphasis	−0.032685781
Wavelet-LLH	Firstorder	Skewness	−0.024816826
Wavelet-HLL	Glszm	GrayLevelNonUniformityNormalized	0.069964718
Log-sigma-5-0-mm-3D	Glcm	Imc1	−0.006757529
Wavelet-LLL	Ngtdm	Coarseness	0.059664695

**Table 3:** Comparison between the HU and Rad score in the osteoporosis and non-osteoporosis subject groups

	Osteoporosis		Non-osteoporosis		*p* value
	Mean and SD	95% CI	Mean and SD	95% CI	
HU	80.77 ± 33.94	74.82–86.74	146.19 ± 50.02	140.07–152.31	< 0.01
Rad score	0.62 ± 0.18	0.59–0.65	0.18 ± 0.22	0.15–0.21	< 0.01

### Evaluation of the HU and radiomics signature models’ performance

**Fig. 4:**
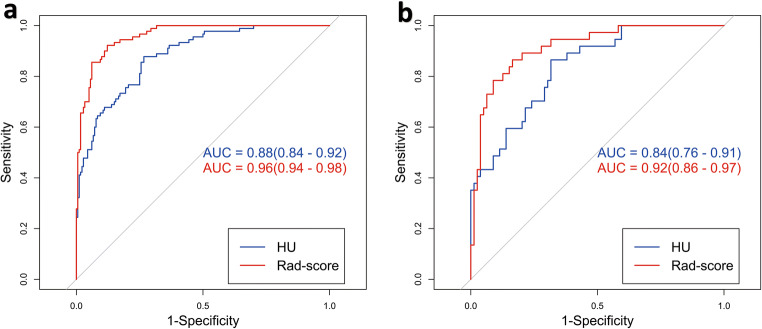
ROC analysis showing that the performance of the radiomics signature model (red line) was better than that of the HU model (blue line) in both the training (**a**) and test (**b**) cohorts

**Fig. 5:**
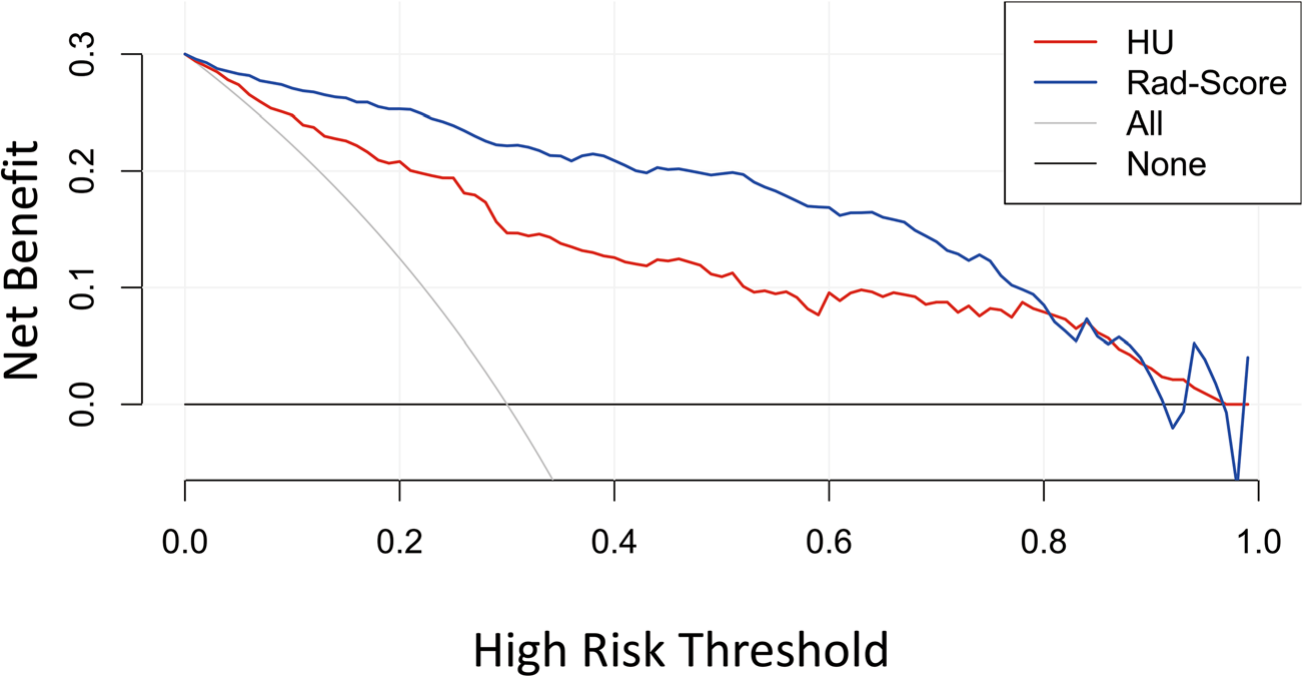
DCA revealed that the Rad score model (blue line) was more advantageous than the HU model (red line). The x axis corresponds to the threshold probability while the y axis corresponds to the net benefit. The gray line represents the assumption that all lesions are due to osteoporosis. The black line indicates that no lesion is due to osteoporosis

The ROC curves of the HU and radiomics signature models are illustrated in Fig. 4. The DeLong test showed that the radiomic signature model was more effective than the HU model in predicting osteoporosis. In the training set, the AUC values for the radiomic signature and HU models were 0.960 and 0.883, respectively (*p* < 0.05), while in the test set, they were 0.915 and 0.836, respectively (*p* < 0.05) (Table 4). Based on the DCA, the radiomics model outperformed the HU model (Fig. 5).

**Table 4:** Performance of the HU and radiomic signature models

	Training cohort				Test cohort			
	SEN	SPE	ACC	AUC (95% CI)	SEN	SPE	ACC	AUC (95% CI)
HU	0.656	0.894	0.815	0.842–0.923	0.595	0.835	0.759	0.762–0.910
Radiomics	0.822	0.939	0.900	0.940–0.980	0.730	0.937	0.871	0.862–0.969

*SEN*, sensitivity; *SPE*, specificity; *ACC*, accuracy; *AUC*, area under the curve; *95% CI*, 95% confidence interval

## Discussion

The present study demonstrates that a radiomic signature model based on preoperative lumbar spinal CT can be used to diagnose osteoporosis. In both training and test sets, the radiomic signature model showed excellent prediction performance. Moreover, the radiomic signature model was more efficient for detecting osteoporosis than the HU model, based on AUC and the DCA curve. A positive result from the radiomic model could alert physicians to perform additional DEXA to confirm the presence of osteoporosis. In contrast, further testing and treatment may be unnecessary when the radiomic model shows a low probability of osteoporosis. This method may decrease medical costs and radiation exposure.

In the radiomic signature model development, 846 candidate radiomic features with ICCs > 0.75 were reduced to 12 features by combining the mRMR and LASSO methods. Combining multiple imaging features in the radiomic signature model can successfully stratify patients into low- or high-score groups with significantly different probabilities of osteoporosis. Additional examinations or treatment should be considered in patients with a higher probability of osteoporosis. Some selected features, such as skewness in the first-order feature class, appear to be independently related to osteoporosis; however, it is challenging to reliably correlate a single feature with the pathological state [17]. Therefore, constructing a multi-feature model is a more feasible approach for osteoporosis screening [18].

Osteoporosis is one of the most important factors associated with certain complications after spinal fusion surgery, including screw loosening, non-union, and cage subsidence; surgeons should be aware of its presence in patients. Some elective spine surgeries could be postponed after pharmacological intervention for osteoporosis. Bone mineral density can be derived from quantitative CT (QCT), ultrasound, and DEXA. However, the World Health Organization defines osteoporosis solely based on DEXA measurements. According to the International Society for Clinical Densitometry (ISCD) recommendations, the DEXA measurement should include the spine and hips, and osteoporosis should be diagnosed based on the lowest *T*-score between the spine and hips. QCT, which focuses on the cancellous bone, can be used to determine bone quality. The clinical application of QCT is often hindered by high economic costs and the required specific training [19]. As QCT is not routinely applied in our hospital, we could not use QCT images in this retrospective study.

At present, HU measurement is still the most frequently used method for opportunistic osteoporosis screening; therefore, it is reasonable to compare the radiomic method to the HU method. Da Zou et al [20] used the HU value to screen osteoporosis in patients with lumbar degenerative disease with 88.5% specificity and 60.8% sensitivity. Cohen et al [21] performed a validation study of opportunistic osteoporosis screening with routine CT on a multiethnic Middle Eastern population. The sensitivity obtained was 76%, and the specificity was 74%. The efficacy of the HU model in this study is similar to that of previous studies. Furthermore, both the Delong test and DCA analysis showed that the radiomics model outperformed the HU model.

Radiomics analysis based on routine preoperative lumbar CT scans could provide an alternative for bone health screening. However, this method cannot replace DEXA, which is still the standard examination recommended by the ISCD. Nonetheless, our method could alert spine surgeons to investigate and possibly treat osteoporosis. Currently, the procedure for radiomics analysis may seem complex; however, the progress of automated segmentation might allow the integration of the feature extraction and calculation into a software program. The radiomics method will eventually become a one-button operation. The present study demonstrated that radiomics analysis based on lumbar spine CT scans might be an effective method to screen for osteoporosis before surgery.

The study has some limitations. First, it was a retrospective study performed at one institution. Further prospective studies with larger sample sizes are warranted. Second, we only focused on the correlation between the radiomic signature and DEXA results, which may not reflect the risk of osteoporosis-related complications, such as pseudarthrosis after lumbar spinal fusion. Finally, the radiomic method requires additional software operations compared to the HU method. Nonetheless, we believe that the radiomic method is worthwhile because it includes more imaging information, such as the cortical bone. The radiomic features extracted from vertebral bodies may be useful for analyzing other osteoporosis-related diseases.

In conclusion, we developed and validated a CT-based radiomic signature model to detect osteoporosis before lumbar spinal surgery. The radiomic method may provide valuable information and facilitate surgical decision-making without additional medical costs and radiation exposure.

## Supplementary Information

ESM 1 (CSV 4711 kb)

ESM 2 (XLSX 1341 kb)

ESM 3 (CSV 55 kb)

## Abbreviations

3D: Three-dimensional
BMD: Bone mineral density
DCA: Decision curve analysis
DEXA: Dual-energy X-ray absorptiometry
ICC: Intraclass correlation coefficient
LASSO: Least absolute shrinkage and selection operator
mRMR: Maximum relevance minimum redundancy
Rad score: Radiomics score

## Declarations

### Guarantor

The scientific guarantor of this publication is Chun-Mei Chen, who is the corresponding author of the article.

### Statistics and biometry

Yan-Wei Jiang, the first author of this manuscript, has significant statistical expertise.

### Methodology

• retrospective

• case-control study

• performed at one institution
